# Novel Intravenous Immunoglobulin Therapy for the Prevention and Treatment of Candida auris and Candida albicans Disseminated Candidiasis

**DOI:** 10.1128/msphere.00584-22

**Published:** 2023-01-23

**Authors:** Hong Xin, Jonothan A. Rosario-Colon, Karen Eberle

**Affiliations:** a Louisiana State University Health Sciences Center, Department of Microbiology, Immunology, and Parasitology, New Orleans, Louisiana, USA; University of Georgia

**Keywords:** adjunctive immunotherapy, *Candida albicans*, *Candida auris*, IVIG, antibody function, disseminated candidiasis, multidrug resistance

## Abstract

Disseminated candidiasis is a life-threatening disease and remains the most common bloodstream infection in hospitalized patients in the United States. Despite the availability of modern antifungal therapy, the crude mortality rate in the last decade has remained unacceptably high. Novel approaches are urgently needed to supplement or replace current antifungal therapies. In our study, we show that human intravenous immunoglobulin (IVIG) can provide protection against Candida auris and Candida albicans disseminated infections in A/J and C57BL/6 mouse models. The protective efficacy of IVIG is evidenced by the prolonged survival of mice with invasive candidiasis that were treated with human IVIG alone or in combination with amphotericin B. Our previous studies have led to the identification of a panel of *Candida* cell surface peptide and glycan epitopes, which are targeted by protective mouse monoclonal antibodies (mAbs) against invasive candidiasis. Of interest, the peptide- and glycan-specific IgGs could be detected in all 18 human IVIG samples. In particular, the specific IVIG lots with the highest protective peptide- and glycan-related IgGs provided the best protection. The combination of IVIG and amphotericin B had enhanced efficacy in protection compared to monotherapy against both multidrug-resistant (MDR) C. auris and C. albicans, with evidence of significantly prolonged survival and lower fungal burdens in targeted organs. This study provides evidence that the protective effects of IVIG were associated with the protective antibodies found in normal human donor sera against pathogenic *Candida*, and IVIG can be a novel therapy or adjunctive therapy with modern antifungal drugs against disseminated candidiasis.

**IMPORTANCE** Since current antifungal treatments are ineffective in the immunocompromised population and no vaccine is available for humans, hope remains that antibody preparations selected for specific fungal antigens may make it possible to reduce the incidence and mortality of invasive candidiasis. Intravenous immunoglobulin (IVIG) has long been approved as a standard treatment for patients with immunodeficiency disorders who are also susceptible to fungal infection. IVIG has been widely used as prophylaxis or supplemental treatment for sepsis and septic shock; however, this form of adjunctive therapy lacks convincing data to establish its efficacy. In this study, 18 samples from commercial IVIG preparations were screened and evaluated by enzyme-linked immunosorbent assays (ELISAs); *Candida* peptide- and glycan-specific IgGs were detected with various titers among all IVIG lots. Importantly, significantly reduced organ fungal burdens and mortality were demonstrated in IVIG-treated mouse models of invasive candidiasis. IVIG lots with higher titers of *Candida*-specific IgGs provided better protection. These findings are important in (i) selecting *Candida*-specific IVIG therapy that may overcome several shortcomings of conventional IVIG therapy by targeting specific antigens responsible for disease pathogenesis, (ii) enhancing protective efficacy, and (iii) validating data from our previous studies and those of others showing that antibodies combined with conventional antifungal drugs provided enhanced resistance to disease. To our knowledge, this study is the first to demonstrate that human IVIG samples contain protective IgGs targeting *Candida* cell surface antigens and can be a novel therapy or adjunctive therapy with modern antifungal drugs against disseminated candidiasis.

## INTRODUCTION

Disseminated candidiasis is a life-threatening disease and remains the most common bloodstream infection in hospitalized patients in the United States. Despite the availability of modern antifungal therapy, the crude mortality rate in the last decade has remained unacceptably high (1–4). In particular, Candida auris is a multidrug-resistant (MDR) health care-associated fungal pathogen and has emerged as the first fungal pathogen to cause a global public health threat (5). The emergence of this newer species raises concerns for even worsening morbidity and mortality. Currently, no specific therapy or vaccine has been approved for human use.

Our previous studies have produced a panel of protective monoclonal antibodies (mAbs) targeting *Candida* cell surface antigens, which play a critical role in reducing the fungal burden and improving clearance. The identified protective Candida cell surface antigens include the following four peptide epitopes and one glycan epitope (6): fructose-bisphosphate aldolase (Fba), methyltetrahydropteroyltriglutamate 6 (Met6), hyphal wall protein 1 (Hwp1), phosphoglycerate kinase 1 (Pgk1), and β-mannan trisaccharide [β-(Man)_3_]. Indeed, the efficacy of antibodies (Abs) against invasive candidiasis is dependent on epitope specificity, abundance, and isotype (7–9). Intravenous immunoglobulins (IVIGs) are therapeutic products of normal human IgG that have been used for years as a substitutive therapy in patients with primary antibody deficiencies. Recently, IVIGs have been used as anti-infectious agents against viruses and bacteria in both patients and experimental models and as prophylactic and supplemental treatments against sepsis, including fungal sepsis (10–13). However, investigations of IVIG therapy in fungal infections are still very limited, even though evidence has shown the protective role of immunoglobulins in infection and inflammation mediated by several fungal species, including *Candida*, Aspergillus, Cryptococcus, *Rhizopus*, and others (14–16). It is difficult for clinicians to establish an antifungal IVIG protocol due to the lack of quantity standards and consistent data. However, IVIG therapy could be beneficial against invasive *Candida* infections if the antibodies found in normal serum samples are protective against pathogenic *Candida* isolates. The failure of IVIG therapy may reflect insufficient amounts of protective antibodies within the mixture of an enormous antibody pool (14). Since we have identified a panel of conserved *Candida* cell surface epitopes targeted by protective mouse mAbs (6, 7, 17–22), we first investigated if the same epitope-specific protective antibodies also exist in IVIG samples from healthy donors. In the present study, we used well-established mouse models of invasive candidiasis caused by C. auris and C. albicans (23) to determine the efficacy of prophylactic and therapeutic intravenous (i.v.) treatments with human IVIG prepared from healthy donors. By evaluating and comparing epitope-specific IgGs in 18 IVIG lots, IVIG samples with the highest titers of epitope-specific IgGs or relatively lower IgG titers were selected, tested, and compared side by side for *in vivo* efficacy.

Although all IVIG lots contain IgG antibodies specific for these cell surface epitopes, different levels of protection were achieved when lethally challenged mice were treated with different IVIG lots. Expectedly, the survival and recovery of the lethally infected mice were associated with the titers of peptide-specific IgGs, and the best protection was provided by selected IVIG samples containing the highest titers of IgGs specific for all five epitopes. These results indicate that antibodies play an important role in protection and that IVIG can be used as a first-line therapy. Uniquely, the five selected *Candida* cell surface epitopes have high homology among medically relevant non-*albicans Candida* (NAC) species, and IVIG lots containing high-titer specific IgGs also have the potential to protect against human NAC pathogens. Moreover, *Candida* is the most frequent cause of fungal severe sepsis or septic shock in intensive care unit (ICU) patients, which unfortunately leads to unacceptably high mortality rates. The failure of anti-inflammatory therapy, including IVIG infusion, might be due to uncurbed existing inflammation at the time when patients are admitted to the ICU. The prophylactic administration of IVIG containing high-titer protective antifungal IgGs to remove pathogens will be the most efficient way to prevent sepsis, in addition to its anti-inflammatory function.

## RESULTS

### IVIG lots contain IgGs specific for a panel of peptides and a glycan epitope that are targeted by protective mouse mAbs in a mouse model of disseminated candidiasis.

Our previous studies have led to the identification of a panel of *Candida* cell surface peptides and glycan epitopes, which are targeted by protective monoclonal antibodies (mAbs) against invasive candidiasis. IVIG formulations were pooled from plasma samples from large numbers of donors, which ensured that the IgG lots contained comparable levels of antigen-specific antibodies. Eighteen lots of commercially obtained IVIG samples (lots A to R; ADMA Biologics) were screened for IgGs specific for the identified protective epitopes, and antibody endpoint titers were determined by enzyme-linked immunosorbent assays (ELISAs) (Table 1). Not surprisingly, specific IgGs recognizing the five selected epitopes were detected in all 18 lots of IVIG. ELISA data demonstrated that there are substantial lot-to-lot differences in the epitope-specific IgG antibody levels, and 18 IVIG samples showed a wide range of endpoint titers from 2,000 to 512,000 (Table 1). For example, IVIG lot A (batch 1741) contains the highest titers of IgGs against all five epitopes; lot P (batch 1743) has titers of IgGs for four epitopes comparable to those of lot A, except that it contains 16-fold-lower titers of IgGs to the Fba peptide; and lot C has the lowest titers to both the Fba and Met6 peptides among 18 samples. On the other hand, lot K (batch 1499) contains the lowest titers to all epitopes, and lot B contains comparably high titers of specific IgGs except for 4-fold-lower titers for trimannose, the glycan epitope. In conclusion, our identified *Candida* cell surface epitopes can be used to select IVIG batches with relatively high titers of potentially protective anti-*Candida* IgGs.

**TABLE 1:** IVIG lots containing IgGs specific for a panel of peptides and a glycan epitope that are targeted by protective mouse mAbs in mouse models of disseminated candidiasis^*a*^

IVIG lot	Titer against *Candida* cell surface or glycan epitope				
	Fba^*b*^	Met6^*c*^	β-1,2-Mannotriose^*d*^	Hwp1^*e*^	Pgk1^*e*^
A (batch 1741)	512,000	16,000	32,000	16,000	6,400
B	128,000	16,000	8,000	16,000	6,400
C	4,000	2,000	32,000	16,000	6,400
D	32,000	2,000	8,000	8,000	6,400
E	4,000	2,000	8,000	2,000	4,000
F	4,000	2,000	8,000	2,000	4,000
G	64,000	2,000	32,000	8,000	64,000
H	4,000	2,000	8,000	2,000	4,000
I	4,000	2,000	8,000	2,000	4,000
J	32,000	4,000	8,000	8,000	4,000
K (batch 1499)	4,000	2,000	8,000	2,000	4,000
L	4,000	4,000	8,000	2,000	4,000
M	8,000	2,000	8,000	8,000	4,000
N	4,000	2,000	8,000	2,000	4,000
O	4,000	2,000	8,000	2,000	4,000
P (batch 1743)	32,000	16,000	32,000	8,000	6,400
Q	4,000	2,000	8,000	2,000	4,000
R	4,000	2,000	8,000	4,000	4,000

a A panel of *Candida* cell surface peptides and a glycan epitope were identified and targeted by protective mAbs against invasive candidiasis in mice. Eighteen lots of commercially obtained IVIG samples (lots A to R; ADMA Biologics) were screened for IgGs specific for the identified protective epitopes, and antibody endpoint titers were determined by ELISAs. Specific IgGs recognizing the five selected epitopes were detected in all 18 lots of IVIG. ELISA data demonstrated that there are substantial lot-to-lot differences in the epitope-specific IgG antibody levels. Fba, fructose bisphosphate aldolase; Hwp1, hyphal wall protein 1; Pgk1, phosphoglycerate kinase 1; Met6, methyltetrahydropteroyltriglutamate 6.

b See references 15–18.

c See references 15, 17, 18, and 21.

d See references 6, 19, and 20.

e See references 8 and 15.

### We have established mouse models of intravenous disseminated infection by C. auris in addition to C. albicans.

C. albicans is the most common disease-causing species (65%); however, the prevalence of disease caused by non-*albicans Candida* (NAC) species is on the rise, along with an increase in antifungal drug resistance. Knowledge of immunity to NAC species is still at an early stage due to the lack of tractable animal models since many NAC species are not usually pathogenic in mouse models of candidiasis. We are the first group that established an A/J mouse (C5 deficiency) model of systemic C. auris infection without immunosuppression (23), and here, we have also established C57BL/6 and BALB/c models of C. auris infection with immunosuppression (see Table 3). The established mouse models closely mimic immunocompromised patient situations and are valuable tools for evaluating the *in vivo* efficacy of mAb and antifungal immunotherapies. Briefly, for disseminated C. auris infection in C57BL/6 and BALB/c mice, a combination of a high inoculum and immunosuppression (dose of 200 mg/kg of body weight of cyclophosphamide [CY] by intraperitoneal [i.p.] administration given for the first dose and then 150 mg/kg given weekly) was enough to establish severe acute infection.

### Human IVIG provided preventive protection against C. auris and C. albicans disseminated infections in mouse models.

**FIG 1:**
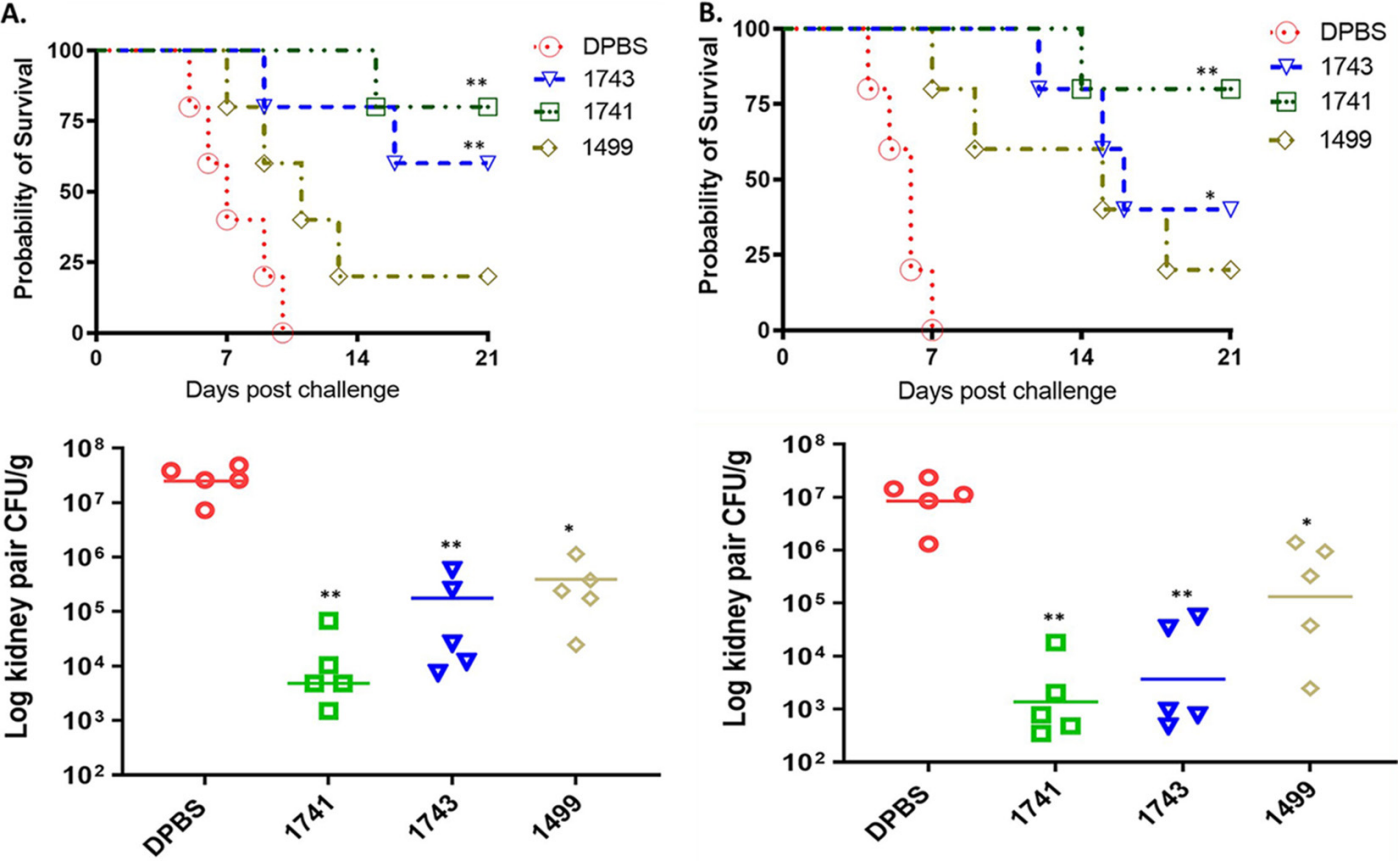
Passive transfer of human IVIG samples from healthy donors can provide protection against disseminated C. auris and C. albicans infections in mice. Based on the endpoint titers of specific IgGs, we first investigated the preventative efficacy of three IVIG lots in protection against C. auris disseminated infections in an A/J mouse model (A) and C. albicans disseminated infections in an immunosuppressed C57BL/6 mouse model (200-mg/kg dose of cyclophosphamide intraperitoneally [i.p.] given weekly) (B). We performed i.p. injections of each selected IVIG lot into mice 4 h before lethal intravenous challenge with C. auris (AR-CDC0386, a human clinical isolate from South American clade IV) (2 × 10^8^ CFU) or C. albicans (SC5314) (1 × 10^6^ CFU) a reference strain, which is a clinical isolate. IVIG batch 1741, containing the highest IgG titers against all five *Candida* cell surface epitopes, provided the best protection (60 to 80% survival), with significantly prolonged survival and greatly reduced CFU against both C. auris and C. albicans (*P* < 0.01). Data are means and SD (*n* = 5). A log rank (Mantel-Cox) test and a two-tailed *t* test were used to identify significant differences (**, *P *< 0.01; *, *P *< 0.05). For *Candida* invasive infection, A/J mice (female, 5 to 7 weeks of age) or C57BL/6 mice (female, 5 to 7 weeks of age) were treated with DPBS or each IVIG sample 4 h before lethal challenge with each *Candida* isolate. The mice were monitored for survival up to 21 days postchallenge, and CFU were evaluated in the kidney either when mice succumbed to the disease or at termination on day 21 postinfection (p.i.).

The IVIG composition definitely depends on the antibody composition of the donor population, and each lot varies with every indication (24). If IVIG samples are selected randomly as antifungal therapy, this would lead to inconsistent consequences due to variations from thousands of donors. In this study, IVIG lots were first generalized and defined by the endpoint titers of IgGs specific for 5 conserved epitopes (Table 1), which enabled us to select “*Candida*-specific lots” that are enriched with potentially protective IgGs. ELISA data demonstrated that although all IVIG samples contain epitope-specific IgGs, there are substantial lot-to-lot differences in *Candida* cell surface epitope-specific IgG antibody levels. We first selected 3 IVIG lots out of 18 IVIG samples to evaluate and compare *in vivo* efficacies (Table 2). Batch 1741 contains the highest epitope-related IgG endpoint titers, while batch 1499 contains the lowest; IVIG batch 1743 contains high-titer specific IgGs comparable to those of batch 1741 except with low IgG titers to the Fba peptide epitope. To assess relevance to *in vivo* efficacy, the three selected IVIG lots were evaluated for their prophylactic efficacy against each clinical isolate of C. albicans (SC5314) and C. auris (AR-CDC0386) in our established mouse models (Table 3). The group treated with Dulbecco’s phosphate-buffered saline (DPBS) buffer alone served as the negative controls. All three IVIG lots demonstrated prophylactic protection against disseminated candidiasis caused by both C. albicans and C. auris (Fig. 1), and efficacy was associated with the endpoint titers of the epitope-specific IgGs in each lot. Briefly, each of the 3 selected IVIG lots was transferred to naive mice 4 h before lethal challenge with C. auris or C. albicans, survival was observed up to day 21 postinfection (p.i.), and the fungal burden in the kidney (CFU) was analyzed. Our results show that in the A/J mouse model of C. auris invasive infection, the best protection was provided by IVIG batch 1741, containing the highest titers of peptide and glycan epitope-specific IgGs. IVIG batch 1499, which contained the lowest peptide- and glycan-related IgG titers, provided 20% protection, whereas no mice that received the vehicle control survived, succumbing to the disease within 5 to 10 days (Fig. 1A). Consistently, groups that received batch 1743 or 1741 had significantly reduced CFU in their kidneys compared to those in the control and batch 1499-treated groups. Similar *in vivo* efficacy results were obtained with the immunosuppressed C57BL/6 mouse model of C. albicans invasive infection (Fig. 1B). Our data demonstrate that human IVIG contains IgGs targeting the same peptide/glycan epitopes recognized by protective mouse mAbs and that the IVIG lot with the highest titers of protective IgGs provides the best protection against disseminated candidiasis in mice. In reality, manufacturers of IVIG are not required to quantify the specific antibody contents in their preparations. Hence, clinical practitioners cannot be confident of the quantities of pathogen-specific antibodies present in any given production lot of IVIG. Therefore, our identified epitopes might offer a standard to evaluate and quantify specific anti-*Candida* IgG contents, which can help practitioners select IVIG lots that could provide better protection against invasive *Candida* infections in patients. More lots need to be tested, and more data are needed to establish a defined titer threshold that is protective.

**TABLE 2:** Five IVIG lots with various specific IgG endpoint titers selected for *in vivo* efficacy evaluation

IVIG lot	IgG endpoint titer for *Candida* cell surface peptide or glycan epitope^*a*^				
	Fba	Met6	β-1,2-Mannotriose	Hwp1	Pgk1
A (batch 1741)	512,000	16,000	32,000	16,000	6,400
P (batch 1743)	32,000	16,000	32,000	8,000	6,400
K (batch 1499)	4,000	2,000	8,000	2,000	4,000
B	128,000	8,000	8,000	16,000	6,400
C	4,000	2,000	32,000	16,000	6,400

a ELISA results show the endpoint titers of human IgG antibodies of IVIG lots specific for a panel of peptide and glycan epitopes that were targeted by protective mAbs in mouse models of disseminated candidiasis.

**TABLE 3:** Acute *Candida* invasive infection in mouse models^*b*^

*Candida* strain	Challenge dose(s) (CFU)^*a*^	Mouse strains
C. albicans SC5314 (ATCC MYA-2876)	1 × 10^6^, 5 × 10^5^	C57BL/6, BALB/c
C. auris AR-CDC0386	2 × 10^8^	A/J and C57BL/6 (CY i.p. treatment)
C. auris AR-CDC0387	1 × 10^8^	A/J and C57BL/6 (CY i.p. treatment)

a The optimal dose of each *Candida* strain for producing an acute infection with 60 to 100% of animals dying within 10 to 20 days using C57BL/6, BALB/c, and A/J mouse strains.

b We established an inbred A/J (C5^−/−^) mouse model of acute systemic C. auris infection without immunosuppression. Furthermore, an acute C. auris invasive infection model in immunosuppressed C57BL/6 mice was also established. A combination of a large inoculum and immunosuppression (150-mg/kg dose of cyclophosphamide [CY] i.p. given weekly) was enough to establish invasive infection with C. auris in C57BL/6 mice.

### Therapeutic effect of a single injection of intravenous immunoglobulin associated with endpoint titers of epitope-specific IgGs.

**FIG 2:**
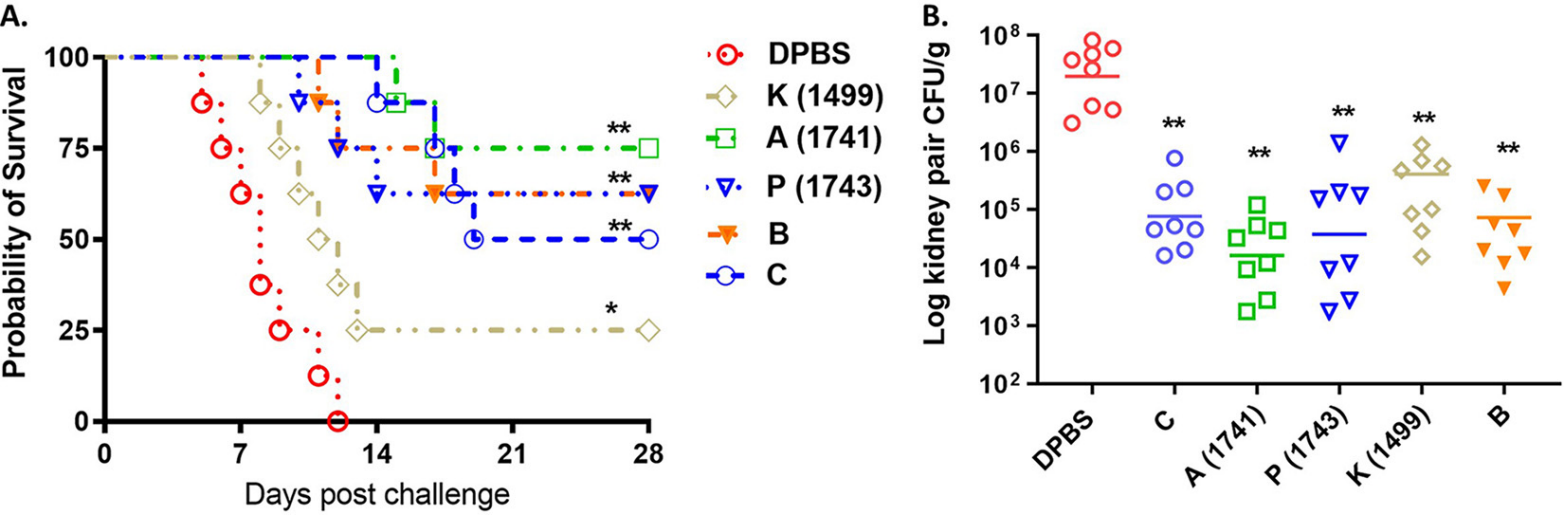
Passive transfer of human IVIG samples from healthy donors can provide therapeutic efficacy against disseminated C. auris invasive infection in mice. We further investigated the therapeutic efficacy of five IVIG lots in protection against C. auris disseminated infections in an A/J mouse model. We performed i.p. injections of each selected IVIG lot into mice 12 h after lethal intravenous challenge with C. auris (AR-CDC0387, a different human clinical isolate from South Asia clade I) (1 × 10^8^ CFU) yeast cells. IVIG batch 1741, containing the highest IgG titers against all five *Candida* cell surface epitopes, provided the best protection (75% survival), with significantly prolonged survival (A) and greatly reduced CFU (B) against C. auris lethal infection compared to the control (*P* < 0.01). The other four lots of IVIG all provided different levels of protection and significantly reduced fungal burdens (*P* < 0.01) in the kidney, which were associated with the specific IgG endpoint titers to the defined protective epitopes. Data are means and SD (*n* = 8). A log rank (Mantel-Cox) test and a two-tailed *t* test were used to identify significant differences (**, *P* < 0.01; *, *P* < 0.05). For *Candida* invasive infection, A/J mice (female, 5 to 7 weeks of age) were treated with DPBS or each IVIG lot 12 h after lethal challenge. The mice were monitored for survival after infection up to 28 days p.i., and CFU were evaluated in the kidney either when mice succumbed to the disease or at termination on day 28 p.i.

Although all IVIG samples demonstrated the presence of protective antibodies, specific peptide-related IgGs varied between individual lots, and these IVIG lots also differed in protective efficacy by passive immunity against invasive candidiasis in mice. We further selected 5 IVIG samples from 18 lots (Table 2). Lot A (batch 1741) has the highest titers of IgGs for all epitopes. Lot P (batch 1743) has high titers for the 4 epitopes comparable to those of lot A except that it has a 16-fold-lower IgG titer to the Fba peptide. Lot K (batch 1499) shows the lowest IgG titers for all epitopes among the 18 lots. Lot B has the lowest titers of glycan-specific IgG, and lot C contains the lowest titers to both peptides Fba and Met6. The selected IVIG lots were evaluated for their *in vivo* therapeutic efficacy against each clinical isolate of C. albicans and C. auris in our established mouse models (Table 3). We have shown that IVIG lots with high-titer IgGs specific for *Candida* cell surface epitopes reduced organ fungal burdens and significantly prolonged survival in mice (Fig. 1). Here, we also started with the same simple means of administration as a single i.p. dose given 12 h after C. auris lethal challenge to evaluate the therapeutic efficacy of IVIGs in mice. In the A/J mouse model of C. auris invasive infection, the therapeutic effects of single treatments with 5 IVIG lots were compared side by side (Fig. 2). Lot A contained high-titer IgGs specific for all five epitopes, providing the best protection among the 5 samples. Lot B, while lacking high titers of glycan-specific IgGs, provided efficacy comparable to that of lot P, which has only low titers of Fba-specific IgGs. IVIG lot C, with low IgG titers to both Fba and Met6, provides moderate protection, with less efficacy than lots A, P, and B. All IVIG lots are more protective than lot K, which is the sample containing the lowest IgG titers for all epitopes (Fig. 2A). Consistent with the survival rates, groups that received batch 1743 or 1741 had greater reductions of CFU in their kidneys than the groups treated with lots K, B, and C. All groups treated with IVIG had significantly reduced fungal burdens compared to the control (Fig. 2B). This study indicates that IVIG batches contain protective IgG antibodies targeting *Candida* cell surface epitopes, and the titers and specificity of these IgGs are associated with protective activity against *Candida* invasive infection.

### IVIG can enhance therapeutic efficacy when combined with conventional antifungal drugs against C. auris and C. albicans invasive infections.

**FIG 3:**
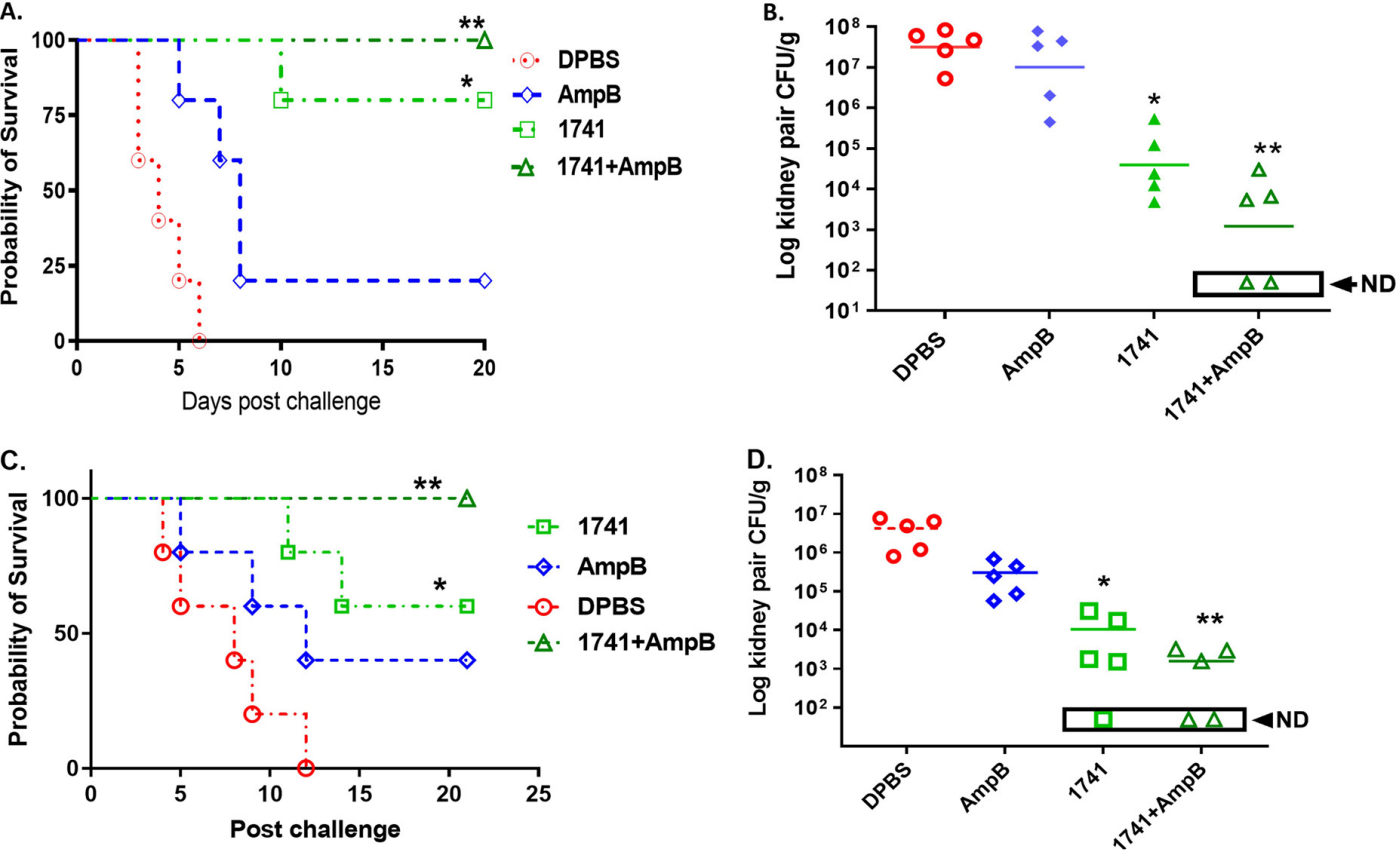
The antifungal activity of amphotericin B (AmpB) against disseminated candidiasis is enhanced in the presence of human IgGs (IVIG). We evaluate the *in vivo* efficacy of combined treatment with AmpB and IVIG batch 1741 containing the highest titers of epitope-specific IgGs. We performed the passive transfer of IVIG batch 1741, AmpB, or AmpB plus batch 1741 to naive mice 12 h after lethal challenge with C. auris AR-CDC0386 (2 × 10^8^ CFU) in A/J mice (A and B) and C. albicans SC5314 (1 × 10^6^ CFU) in immunosuppressed C57BL/6 mice (C and D). (A and B) For the A/J mouse model of disseminated C. auris infection, the group receiving combined treatment with batch 1741 plus AmpB had 100% survival (A) and significantly reduced or nondetectable (ND) CFU in the kidney (B). (C and D) Similarly, in the immunosuppressed C57BL/6 mouse model of disseminated C. albicans infection, the group that received combined treatment with batch 1741 plus AmpB also had 100% survival (C) and significantly reduced or undetectable (ND) CFU in the kidney (C and D). In brief, for *Candida* invasive infection, 4 groups of A/J mice (female, 5 to 7 weeks of age) or C57BL/6 mice (female, 5 to 7 weeks of age) were treated with DPBS, AmpB (minimal effective dose of 0.5 mg/kg), IVIG batch 1741 (200 μL; 5 mg/mL), or IVIG batch 1741 plus AmpB 12 h after lethal challenge with each *Candida* isolate as described in the text. The mice were monitored for survival up to 21 days postinfection, and CFU were evaluated in the kidney either when mice succumbed to the disease or at termination on day 21 p.i.

In clinical situations, the antibody-based immunoprotective approach is more likely to be used in combination with standard antifungal therapy. Given that a single IVIG treatment provided significant prophylactic/therapeutic efficacy against disseminated candidiasis in murine models, we further examined the therapeutic efficacy of IVIG batch 1741 combined with amphotericin B (AmpB) given after infection with C. auris. The survival data showed that the administration of IVIG batch 1741 with AmpB to A/J mice at 12 h p.i. enhanced the therapeutic efficacy of AmpB, leading to a more effective outcome than that with monotherapy with either IVIG or AmpB, evidenced by significantly prolonged survival and reduced fungal burdens (Fig. 3A and B). In a parallel experiment, immunosuppressed C57BL/6 mice treated with IVIG batch 1741 combined with AmpB 12 h after lethal challenge with C. albicans showed similarly enhanced protective efficacy compared to monotherapy, which was evidenced by the significantly prolonged survival and reduced/nondetectable CFU in the kidneys (Fig. 3C and D). We also obtained similar results in the BALB/c mouse model of invasive candidiasis (data not shown). Therefore, the combination of IVIG batch 1741 and AmpB had the greatest efficacy in protection against both C. auris and C. albicans invasive infections in mice, and IVIG enhanced the therapeutic efficacy of AmpB by lowering the drug dosage to reach greater efficacy.

## DISCUSSION

We defined a panel of C. albicans cell surface epitopes in previous studies. Depending on the specificity of the epitope, the related antibodies are protective or nonprotective or even enhance disease (6). Here is a fascinating fact obtained from our anti-*Candida* vaccine and antibody studies: the new concept of immunodeficiency or antibody deficiency is not necessarily related to a lack of antibodies but is related to a lack of protective antibodies. Furthermore, numerous studies demonstrated that natural antibodies have an important role in defense against fungal infections, including *Candida* infections (25–28). In humans, *Candida* is a commensal member of the microflora on mucosal surfaces until the host becomes immunodeficient, so even without *Candida* infection, humans tend to have antibodies against this fungus. In this study, we show that *Candida* surface antigen-specific IgG antibodies were detected among IVIGs from pooled plasma from healthy donors, with high and low levels of specific IgGs in different IVIG lots. The prophylactic or therapeutic benefits of IVIG in this context were initially traced to the ability of IVIG treatment to deliver specific antibodies to recipients who are unable to produce them, particularly antibodies that protect against disease. The reported protective function of IVIG is in line with our *in vivo* efficacy data here, where immunosuppressed mice receiving IVIG lots with high-titer IgGs specific for protective *Candida* epitopes have significantly lower levels of CFU in the kidney and enhanced resistance to invasive candidiasis than those treated with lower-titer specific IgGs. Therefore, this indicated that the administration of IVIG to A/J or immunosuppressed C57BL/6 mice restricted fungal growth and increased survival, thus confirming that IVIGs have an important role in the clearance of pathogens. The efficacy of IVIG seems to be dependent on both the epitope specificity and the abundance of protective antibodies in each sample. The IVIG composition definitely depends on the antibody composition of the donor population, and if IVIG samples are selected randomly as antifungal therapy, this would lead to inconsistent consequences due to variations from thousands of donors. In this study, IVIG lots were defined by the endpoint titers of IgGs specific for a panel of conserved *Candida* protective epitopes, which makes generalization possible and ensures the selection of anti-*Candida*-specific lots that are enriched with potentially protective IgGs. Hence, our specific epitopes can be used to select IVIG lots with high titers of anti-*Candida* protective IgGs to obtain greater efficacy for prophylactic or therapeutic purposes. IVIG can be more effective in treating infections with MDR *Candida* species than conventional antifungal drugs since IVIG containing high concentrations of IgGs specific for the *Candida* antigen will not be expected to select for drug-resistant *Candida* species. The approach of using such *Candida*-specific IVIG lots is similar to the approach using so-called “hyperimmune preparations,” i.e., IgGs collected from donors with high titers of the desired antibodies. Moreover, such therapeutic IVIGs selected against multiple *Candida* species could even be superior to hyperimmune therapy, which is usually active against only one specific pathogen. As a treatment strategy for immunocompromised patients, the passive transfer of IVIG with high-titer protective anti-*Candida* IgGs can provide instant protection and leads to the efficient clearance of specific pathogenic microorganisms, thus reducing mortality. Other studies also implied that prophylaxis or therapeutic treatment with IgG might be beneficial if sufficient levels of agent-specific antibodies are present. For example, monthly prophylaxis significantly reduced the severity of respiratory syncytial virus (RSV) infections in very young high-risk patients (29–31). Furthermore, to enhance the efficacy of IVIG therapy, a multidose regimen can be used; for example, *Candida*-specific IVIG may be given every month to increase efficacy, especially in cases of immunocompromised and immunosuppressed patients.

The advantages and disadvantages of antibody-based therapies are often compared with those of conventional antimicrobial drugs. We have developed a panel of anti-*Candida* protective monoclonal antibodies (mAbs) targeting *Candida* cell surface epitopes; the potential of mAb therapy is vast, especially for *Candida* species that are resistant to conventional antibiotics. Here, we further demonstrated that IVIG lots with high-titer IgGs against the protective epitopes were sufficient to provide protection in an immunosuppressed setting by a single infusion. Hence, the ultimate goal of our Ab work is to provide immediate protection by the passive administration of IVIG or mAbs, representing a strong adjunctive measure for conventional antifungal drug therapy. mAbs inherently have high specificity for the pathogen without selecting for drug resistance. However, a disadvantage of high specificity is the emergence of variants that lack the determinant that the antibody recognizes. The use of mAb cocktails targeting several antigens could obviate this concern. However, this approach would have the drawbacks of an increased cost as well as the concerns and regulatory issues involving efficacy and safety. On the other hand, FDA-long-approved IVIGs used as therapeutic reagents have the advantages of the enormous diversity of specificities and several isotypes as well as being much less expensive than mAb products. Although serum IgG antibodies may promote natural resistance to infection, this may not be sufficient to prevent disseminated infection. Our study data show for the first time that human IgGs target the same protective peptide/glycan epitopes as protective mouse mAbs and can confer protection in mouse models of disseminated candidiasis. Therefore, the identified *Candida* cell surface epitopes can be used to select IVIG batches with relatively high titers of protective anti-*Candida* IgGs to obtain effective therapeutics against the disease. In particular, in this study, we selected IVIG batch 1741, with the highest titers of antigen-specific IgGs, and evaluated its function in enhancing AmpB efficacy compared to that of batch 1741 or AmpB as an antifungal alone. We chose the minimal therapeutic dose of AmpB that was effective against both the C. auris AR-CDC0386 and C. albicans SC5314 isolates in our previous studies and investigated the potential of IVIG as an adjunctive therapy to enhance AmpB’s *in vivo* efficacy. Our data showed that IVIG batch 1741 combined with AmpB not only increased survival significantly but also cleared fungi from the targeted organs more efficiently. Hence, combined with or as an adjunctive therapy along with conventional antifungal agents, anti-*Candida* IVIG therapy can enhance host resistance and clear fungal pathogens even more efficiently.

In addition to targeting protective epitopes conserved across *Candida* species, *Candida*-specific IVIG therapy can exert its action through nonspecific interactions with various immune molecules and cells. Hence, IVIG can also play a regulatory role in the activation of innate immune cells by signaling via diverse Fc receptors for anti-inflammatory and immunomodulatory effects (14, 32–34). IVIG treatment was reported to reduce intestinal inflammation in mice and to eliminate C. albicans overgrowth from the gut in association with the downregulation of proinflammatory mediators combined with the upregulation of anti-inflammatory cytokines (35). Therefore, the clinical rationale for the use of IVIG therapy in fungal infection and fungal sepsis can be both the roles of immunoglobulins in the clearance of pathogens and immunomodulation (14).

### Conclusion.

This study highlights the importance of anti-*Candida* human IgG in the circulation in mediating resistance to *Candida* invasive infections. The ability of IVIG lots to provide high-level protection in our immunocompromised mouse model also suggests that pathogen-specific human IgG antibodies can be effective in patients with immune defects. Our selected *Candida*-specific IVIG lots are aimed at overcoming several shortcomings of conventional IVIG preparations by selecting high-titer IgGs targeting protective cell surface antigens of pathogenic *Candida* isolates. Passive immunization with *Candida*-specific IVIG can be a rapid and effective therapeutic measure in such individuals who are so ill that high-dose toxic antibiotics are not appropriate. Furthermore, an important advantage of antibody therapies is their synergistic or additive role when combined with conventional antimicrobial chemotherapy. Treatment for invasive candidiasis in immunocompromised patients is always challenging in clinical management since modern antifungal chemotherapy is ineffective and often unable to eradicate the infection. In future studies, we will evaluate whether *Candida*-specific IVIG can complement/synergize with the activity of antifungal agents against the disease at even later time points (48 h or 72 h postchallenge) and further investigate if IVIG has synergy/enhanced efficacy when combined with other classes of antifungals such as azoles and polyenes. For more effective therapeutic strategies, it is critical to know if specific IVIG can improve antifungal treatment with greater efficacy by reducing drug doses against MDR C. auris clinical isolates and other drug-resistant *Candida* isolates with high MICs.

The use of IVIG as an adjunctive immunotherapy to enhance the efficacy of antifungal drugs is a promising strategy to improve the prognosis of these patients. Consequently, antibody-based (IVIG) therapies could easily be incorporated into existing treatment protocols. Conclusively, this study offers strong evidence of the preclinical efficacy of therapeutic IVIG and provides compelling data to advance this strategy into the clinic.

## MATERIALS AND METHODS

### Organisms and culture conditions.

C. albicans SC5314 (ATCC MYA-2876), C. auris AR-CDC0387, and C. auris AR-CDC0386 were grown as stationary-phase yeast cells in glucose-yeast extract-peptone (GYEP) broth at 37°C, washed, suspended in cold Dulbecco’s phosphate-buffered saline (DPBS; Sigma), and used to infect mice.

### Mice.

Female C57BL/6 mice (The Jackson Laboratory, Bar Harbor, ME, USA) and BALB/c and A/J mice (Charles River Laboratories), 5 to 7 weeks old, were used throughout the study. Mice were always maintained and handled according to a protocol (protocol number 3710) approved by the Institutional Animal Care and Use Committee (IACUC) at the Louisiana State University Health Sciences Center (LSUHSC) in New Orleans, LA.

### IVIG and pooled plasma preparations.

Human IgG preparations from random healthy donors (IVIG lots A to R [18 samples]) containing 5 mg/mL IgG were obtained as a gift from Jimmy Mond of ADMA Biologics. Among the 18 samples, lots A, G, and K were found to have high-titer anti-RSV IgGs according to information provided by ADMA Biologics. Lot A (batch 1741), lot K (batch 1499), and lot P (batch 1743) were assigned specific batch numbers individually, which were also provided by ADMA Biologics. The plasma was passed over a protein A column and then concentrated/dialyzed against saline. The total milligrams of IgG in ADMA Biologics IVIG samples following sterile filtration were determined, and phosphate-buffered saline (PBS) was added to bring the concentration to 5 mg/mL.

### Synthetic peptide and glycan antigens.

We identified and isolated a panel of protective mouse monoclonal antibodies (mAbs) specific for C. albicans cell surface peptides and a glycan epitope, and each mAb is protective in both immunocompetent mice and neutropenic mice. Therefore, we selected the following four conserved 14-mer protective peptide epitopes and one glycan epitope as targets for human IgGs: Fba (YGKDVKDLFDYAQE), Met6 (PRIGGQRELKKITE), Hwp1, and phosphoglycerate kinase 1 (Pgk1) (VPLDGKTITNNQRI). Each synthetic peptide was produced commercially (GenScript) and used as the coating antigen on enzyme immunoassay (EIA) plates. The purity of each peptide was >98.5%. The glycan epitope β-1,2-mannotriose (gift from David Bundle) was conjugated to bovine serum albumin (BSA) as a trimannose-BSA conjugate for EIA plate coating.

### Immunosuppression.

Five- to seven‐week‐old female C57BL/6 and BALB/c mice were immunosuppressed using cyclophosphamide monohydrate (CY) (catalog number C0768; Sigma‐Aldrich, St. Louis, MO, USA) 3 days prior to challenge by intraperitoneal (i.p.) injection using a dose of 200 mg/kg of body weight. Immunosuppression was maintained with additional i.p. injections of a 150-mg/kg dose of cyclophosphamide every 7 days.

### Evaluation of endpoint titers of antipeptide/antiglycan IgGs of IVIG samples by ELISAs.

Each IVIG lot was analyzed by an enzyme-linked immunosorbent assay (ELISA) for endpoint titers of IgGs specific for each peptide epitope and β-1,2-mannotriose (Table 1). Briefly, β-mannotriose–BSA or synthetic peptides were dissolved at 5 μg/mL in PBS (pH 7.4). The solution of each lot was used to coat 96-well ELISA plates (100 μL/well, overnight at 4°C). Duplicate samples of each IVIG lot were tested, as were DPBS buffer and horseradish peroxidase (HRP)-conjugated anti-human IgG secondary antibody as controls. IVIG samples were serially diluted 2-fold, with a starting dilution of 1:100. The secondary antibody was mouse anti-human IgG conjugated to horseradish peroxidase (catalog number A5420; Sigma) (diluted 1:5,000 in PBS plus Tween [PBST]), 100 μL of which was added to each well, and the plates were incubated for 1 h at 21°C and washed, followed by the addition of 100 μL of the substrate solution (25 mL of 0.05 M phosphate-citrate buffer [pH 5.0], 200 μL of an aqueous solution of *O*-phenylenediamine at 50 mg/mL [Sigma], and 10 μL of 30% H_2_O_2_). The color was allowed to develop for 10 min, the reaction was stopped by the addition of 100 μL of 2 M H_2_SO_4_, and the absorbance was read at 492 nm (microtiter plate reader, model 450; Bio-Rad, Richmond, CA). The background absorbance from the negative-control wells was subtracted from the absorbance of the test well to obtain final optical density (OD) readings. The ELISA titer was taken as the reciprocal of the last antibody dilution that gave a positive OD reading, a value two or three times higher than the mean background or negative-control reading. The specificity of each peptide-related IgG response was confirmed by a competition ELISA by using each peptide as an inhibitor to compete for IgG antibody binding sites with solid face-absorbed peptide antigen (data not shown).

### Protection against fatal invasive *Candida* infection by passive transfer of human IVIG.

The aim of our passive-transfer experiments was to examine whether the adoptive transfer of human IVIG samples from healthy donors protects mice from the development of fatal C. auris and C. albicans disseminated infections. In these experiments, we tested the protective efficacy of selected IVIG samples based on the endpoint titer of IgG specific for protective peptide and glycan antigens on the *Candida* cell surface. In particular, we compared the protective potentials of these human preparations with various endpoint titers of peptide/glycan-specific IgGs. Three IVIG lots (A, K, and P) were selected to assess prophylactic efficacy in mouse models of C. auris and C. albicans disseminated infections. Mice were given an i.p. dose of each IVIG lot 4 h before being hematogenously challenged with a lethal dose of *Candida* cells (C. auris AR-CDC0386 at 2 × 10^8^ CFU and C. albicans SC5314 at 1 × 10^6^ CFU). Furthermore, five IVIG samples (lots A, B, C, K, and P) were further evaluated for their therapeutic effect on C. auris invasive infection using single transfers of IVIG. A/J mice were given an i.p. dose of each lot 12 h after being hematogenously challenged with a lethal dose of *Candida* cells (C. auris AR-CDC0387 at 1 × 10^8^ CFU).

### Assessment of the therapeutic efficacy of IVIG combined with a conventional antimicrobial drug against disseminated candidiasis.

We examined the combination therapy of batch 1741 with liposomal amphotericin B (AmpB) (AmBisome), which remains the standard therapy for invasive candidiasis, especially for multidrug-resistant *Candida* isolates that are resistant to both fluconazole and an echinocandin. Although AmpB resistance appears to be uncommon, there is a certain limit to the use of AmpB because this antifungal drug causes severe renal damage. We evaluated the *in vivo* efficacy of combined treatment with AmpB and IVIG batch 1741, which contains the highest titers of IgGs specific for all five epitopes. We performed passive transfer to naive mice 12 h after lethal challenge with C. auris AR-CDC0386 (2 × 10^8^ CFU) in A/J mice or C. albicans SC5314 (1 × 10^6^ CFU) in C57BL/6 mice. Briefly, four groups of A/J mice or immunosuppressed C57BL/6 mice (female, 5 to 7 weeks of age) were treated with DPBS, AmpB (minimal effective dose of 0.5 mg/kg), IVIG batch 1741 (200 μL; 2 mg/mL), or IVIG batch 1741 plus AmpB 12 h after lethal challenge with each *Candida* isolate, as described above. The mice were monitored for survival up to 21 days postinfection, and CFU in the kidney were evaluated either when mice succumbed to the disease or at termination on day 21 p.i.

### Fungal challenge and assessment of IVIG protection.

Both A/J mice and immunosuppressed C57BL/6 mice were treated via one i.p. injection with 200 μL of each selected IVIG sample or DPBS. We performed i.p. injection with IVIG either 4 h before or 12 h after lethal intravenous (i.v.) challenge with live C. auris (AR-CDC0386, 2 × 10^8^ CFU; AR-CDC0387, 1 × 10^8^ CFU) or C. albicans (SC5314, 1 × 10^6^ CFU) yeast cells as described above. All mice were monitored daily for death or the development of a moribund state, at which point they were sacrificed via CO_2_ inhalation. All surviving mice were sacrificed at the conclusion of each study. Protection was evaluated by both animal survival for up to 21 or 28 days and quantification of the numbers of CFU of *Candida* cells in the targeted organ, the kidney.

### Quantification of fungal burdens.

Upon death or the termination of the experiments, the kidneys were extracted from mice and placed on ice, and each organ was either homogenized in DPBS immediately for further processing or stored at −80°C until processing. The homogenate was then serially diluted and plated onto GYEP agar plates containing chloramphenicol. The plates were incubated for 48 h at 37°C, at which time the CFU were quantified. The limit of detection was 50 CFU/g for each organ.

### Statistical analysis.

The statistical significance of differences in the median survival times (MSTs) was calculated by Kaplan-Meier statistical analysis (GraphPad Prism, version 9). Survival data were evaluated by Kaplan-Meier analysis, and statistical significance was calculated using a log rank (Mantel-Cox) test. For fungal burden data, results are expressed as means ± standard deviations (SD), and statistical significance was calculated using a two‐tailed *t* test to compare mAb‐treated groups to the control group. Each study contained five or eight mice per group unless otherwise stated. Significant *P* values are indicated in the figures (*, *P < *0.05; **, *P < *0.01).
